# John Mendelsohn: A visionary scientist, oncologist and leader

**DOI:** 10.18632/genesandcancer.195

**Published:** 2019

**Authors:** Rakesh Kumar, Ferid Murad, Oliver Bogler, Bert W. O'Malley, Gabriel N. Hortobagyi

**Affiliations:** ^1^ Cancer Biology Program, Rajiv Gandhi Centre for Biotechnology, Trivandrum, Kerala, INDIA; ^2^ Department of Human & Molecular Genetics, Virginia Commonwealth University School of Medicine, Richmond, USA; ^3^ Department of Medicine, Hematology-Oncology, Rutgers New Jersey Medical School, Newark, USA; ^4^ Department of Medicine, Stanford University, Palo Alto, CA, USA; ^5^ Palo Alto Veterans Institute for Research (Stanford Affiliated Hospital), Palo Alto, CA, USA; ^6^ ECHO Institute, University of New Mexico, Albuquerque, USA; ^7^ Department of Cellular and Molecular Biology, Baylor College of Medicine, Houston, Texas, USA; ^8^ Breast Medical Oncology, The University of Texas MD Anderson Cancer, Houston, Texas, USA

**Keywords:** growth factor receptors, cetuximab, trastuzumab, targeted therapy, cancer centers

## Abstract

Dr. John Mendelsohn is credited for the concept of targeting the epidermal growth factor receptor (EGFR), providing the first evidence of anticancer activity of antagonist anti-EGFR mAb, and developing the Erbitux (Cetuximab) drug for cancer patients. During his professional journey, Dr. Mendelsohn also helped to build and elevate the status of three cancer cancers, all while touching the lives of cancer patients around the globe. He was a towering figure, and his passing in January 2019 casts a very long shadow over the entire field of cancer research and treatment. Although no one person can ever adequately fill John Mendelsohn's very large shoes, we can all learn by his remarkable example. Here we discuss Dr. Mendelsohn's professional life to spotlight his influence on oncology and also share personal reflections from us and several colleagues: Tony Hunter, Robert A. Weinberg, Robert C. Bast, Raymond Sawaya, David M. Gershenson, Christopher J Logothetis, Stanley R. Hamilton, Mien-Chie Hung, and George M. Stancel. *See related article* Kumar et al. Can Res 2019; 79:4315-4323.

## INTRODUCTION

Earlier this year, the medical community experienced an astounding loss of one of its towering leaders when Dr. John Mendelsohn passed away after a brave fight against cancer. Although Dr. Mendelsohn spent the bulk of his career at three U.S. institutions-University of California, San Diego (UCSD); Memorial Sloan Kettering Cancer Center (MSKCC); and the University of Texas M.D. Anderson Cancer Center (MD Anderson) -the news of his passing reverberated throughout academic hospitals around the world, a testament to the deep impact of Dr. Mendelsohn's legacy. His life work was devoted to advancing treatments and innovations in a dedicated effort to battle cancer, and we are all fortunate that he chose to fight these battles from multiple approaches. Dr. Mendelsohn was at once an expert clinician, a prolific academician and researcher, and a master administrator. He consistently occupied each of these roles with remarkable aplomb during his career. Through his peerless leadership, he made conceptual advances in cancer research, accelerated the development of critical cancer drugs, expanded the mission and services of cancer hospitals, and trained several generations of cancer biologists and other professionals [1]. Taken together, Dr. Mendelsohn's broad legacy in cancer research and treatment cannot be fully captured in writing, but this Commentary attempts to provide an overview of Dr. Mendelsohn's illustrious decades-long career.

## EARLY CAREER – EDUCATION AND UCSD

The promise of Dr. Mendelsohn's career was evident in his impressive educational background. John Mendelsohn graduated *summa cum laude* from Harvard College in 1958, where he was the first undergraduate student of the co-discoverer of DNA James D. Watson who later shared the Nobel Prize. He then accepted a Fulbright Scholarship in Biochemistry and attended the University of Glasgow, and received his M.D. from Harvard Medical School with honors in 1963. He began postgraduate training as a research fellow at the National Institutes of Health, followed by a fellowship in Hematology and Oncology at Washington University, Saint Louis. After accepting a faculty position at the University of California San Diego, Dr. Mendelsohn was sprinting toward one of the most exciting, accomplished and lengthy careers in cancer medicine.

**Figure 1:**
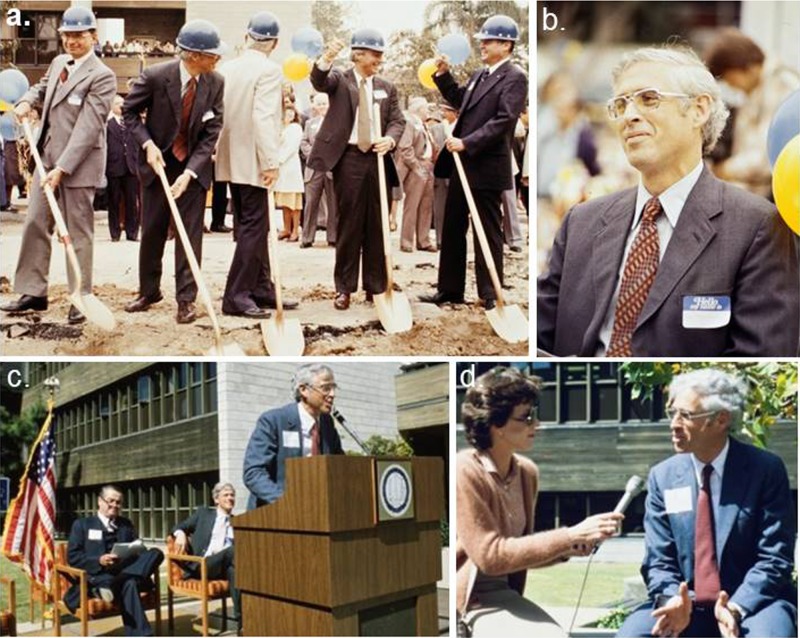
John Mendelsohn – Start of the UCSD Cancer Center **A.** John Mendelsohn at the ground-breaking ceremony at the Theodore Gildred Cancer Center, The University of California San Diego on June 4, 1981. Left to right; Dean John Moxley, John Mendelsohn, William McElroy, Chancellor Richard Atkinson, and George Gildred; **B.** John Mendelsohn with an expression of hope at the event. **C.** John Mendelsohn at the dedication event of the Theodore Gildred Cancer Center, The University of California San Diego on April 15, 1983; and **D.** John Mendelsohn interviewed at the event. (Credit for photos: Courtesy of Special Collections & Archives, University of California San Diego).

At the young age of forty-one, Dr. Mendelsohn was an audacious member of the UCSD faculty and took the first steps in devoting his career to the treatment of cancer. He strongly believed that UCSD needed a center dedicated to cancer care to properly cement its place among premier academic institutions with cancer centers and to most effectively make strides in cancer discoveries and treatment. Through his leadership efforts, UCSD founded its now well-regarded Cancer Center and named Dr. Mendelsohn it's founding Director in 1977, a position he held for nine years. During his tenure, the UCSD Cancer Center succeeded in recruiting talented new faculty starting in 1977, including, a head of Clinical Immunology at the Cancer Center [2], and securing the National Cancer Institute (NCI) Cancer Center designation in 1978 [3]. In addition, Dr. Mendelsohn was instrumental in obtaining $6 million in philanthropic support and NIH funds to complete construction of the UCSD Cancer Center facility, the Theodore Gildred Cancer Center in 1983 (Figure 1). In the words of Mildred Small, the UCSD community felt that the Cancer Center's programs, “have placed San Diego in the forefront of the international war against cancer” [4].

In addition to his transformative administrative successes in creating and leading a Cancer Center at UCSD, John Mendelsohn was equally determined to follow his laboratory interests in growth factor research. Finding a balance between his administrative duties and his passion for bench research, Dr. Mendelsohn began research on his seminal academic work. While collaborating with Dr. Gordon Sato, John Mendelsohn succeeded in translating his well-reasoned EGFR-blocking hypothesis into a molecule of interest, the 225 monoclonal antibody (mAb) [5-10]. In a span of a few years during his UCSD tenure, all of these research efforts culminated in the formation of one of the most powerful hypotheses in cancer medicine and led to the invention of Cetuximab (Erbitux). This period was also the start of targeted receptor-tyrosine kinase-directed cancer therapeutics, which Dr. Mendelsohn was pivotal in developing. Gordon Gills, a UCSD colleague at the time noted that Dr. Mendelsohn was a “builder with a vision” who brought the San Diego “community into the academic world with a shared common enemy: Cancer” [1]. USCD Cancer Center was now on the academic map as one of the leaders in the international war against cancer, and Dr. Mendelsohn was its stalwart leader.

## MSKCC YEARS

The next phase of Dr. Mendelsohn's storied career took him to Manhattan, when in 1985 he joined Memorial Sloan Kettering Cancer Center as the Chair of the Department of Medicine. Utilizing his skilful administrative capabilities, Dr. Mendelsohn worked with Dr. Vincent T. DeVita, Jr. (then the Physician-in-Chief at MSKCC) to create new divisions (including the division of hematologic oncology and solid-tumor oncology) and coordinated a joint mission of the department to advance the mission of developing impactful research. Interestingly, Dr. DeVita – then National Cancer Institute director - was a keynote speaker at the dedication ceremony of Theodore Gildred Cancer facility at UCSD in June 1983.

**Figure 2:**
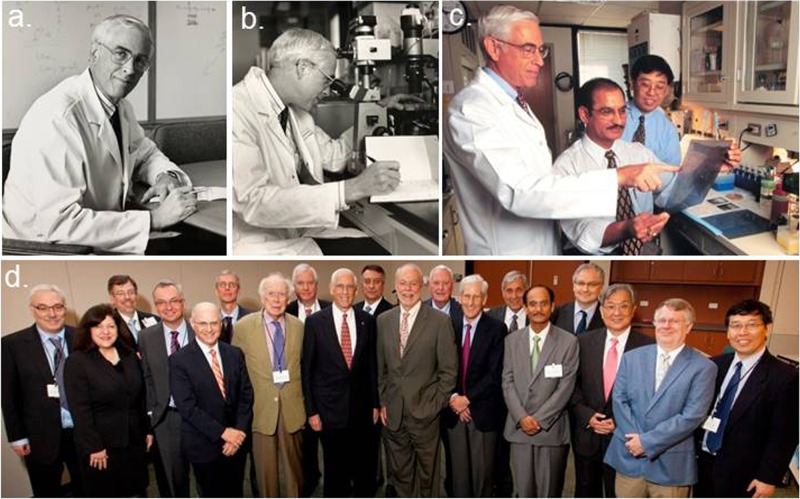
John Mendelsohn's Science and Medicine at MSKCC and MDACC **A.** and **B.** John Mendelsohn in his laboratory seminar room and laboratory at The Memorial Sloan-Kettering Cancer Center; **C.** John Mendelsohn with his colleagues in the laboratory at the University of Texas M.D. Anderson Cancer Center; and **D.** John Mendelsohn with invited colleagues at his Festschrift Symposium on June 21, 2011 to celebrate his lifetime scientific contributions to Cancer research. From left-to-right: Neal Rosen, Margaret Foti, James Abbruzzese, José Baselga, Larry Norton, Giampaolo Tortora, James Watson, Richard O'Reilly, John Mendelsohn, Carlos Cordon-Cardo, Phillip Sharp, Bayard Clarkson, Stuart Kornfeld, Zvi Fuks, Rakesh Kumar, Fortunato Ciardiello, Waun Ki Hong, Gordon Mills, and Zhen Fan. (Credit for photos: A and B, Courtesy of Memorial Sloan-Kettering Cancer Center; C and D, Courtesy of the University of Texas MD Anderson Cancer Center).

Dr. Mendelsohn's relocation to MSKCC provided needed scientific and clinical infrastructure, at that time, to take the anti-EGFR antibodies and pushed forward these discoveries from the bench to the bedside. During this time, Dr. Mendelsohn was intensely focused on understanding the cellular and molecular basis of anti-proliferative properties of anti-EGFR mAbs and developing culture- or mice-based preclinical models [11-14], initiating the first phase I clinical trial of the first anti-EGFR mAb [15], and demonstrating the benefits of combining anti-EGFR mAbs with chemotherapies (Figure 2A, 2B) [16, 17]. Expanding on the principles of EGFR blockage, John Mendelsohn's laboratory also made another significant finding to demonstrate the ability of anti-HER2 mAb 4D5 to inhibit tyrosine phosphorylation [18] and an important milestone in patient care by undertaking pivotal anti-HER2 mAb 4D5 clinical trials along with Larry Norton while at MSKCC, contributing to the development and approval of trastuzumab (Herceptin) by Genentech [19, 20]. The legacy that Dr. Mendelsohn created by this work cannot be understated. As Larry Norton, one of his colleagues at MSKCC, stated: “these ideas have changed clinical practice in so many ways that his legacy as a scientific thinker is vast. But what might be less apparent is how he converted those ideas into clinical trials leading to practical implementation. He did this by-to use the simplest and yet most powerful term - leadership” [1].

## MD ANDERSON YEARS

Powered by his successes and experiences at UCSD and MSKCC and his general desire to do more for cancer patients, John Mendelsohn's leadership of the University of Texas MD Anderson Cancer Center (MD Anderson) was a historic period for our field. Dr. Mendelsohn joined MD Anderson as its third President in 1996, and his visionary leadership transformed and uplifted the institution to new heights. MD Anderson celebrated John's contribution to cancer research and his presidency by organizing the John Mendelsohn's Festschrift, including a scientific symposium, on June 21, 2011 (Figure 2D). Under his leadership from 1996 through 2011, MD Anderson witnessed unprecedented growth, including raising its revenue to $3.1 billion from $726 million, almost doubling the number of employees and total manpower to close to nineteen thousands, growing the MD Anderson's facilities to 15.2 million sq. ft. from 3.4 million sq. ft., and raising MD Anderson's philanthropic support ten-fold to over $2 billion [21-27]. MD Anderson also gained global recognition due to Dr. Mendelsohn's efforts: his creation of the Center for Global Oncology and establishment of twenty sister institutions during his leadership, and created global benefits for MD Anderson's cancer treatment mission [28].

In the midst of leading the institution, John Mendelsohn remained focus in advancing his mission to taking C225 to the bedside. Because of an immense clinical interest in C225 among his colleagues at MD Anderson, Dr. Mendelsohn continued C225's clinical development, leading to its approval by the FDA in 2004 for advanced colorectal cancer patients as a combination with irinotecan, for head and neck cancer in combination with radiotherapy in 2006, and for relapsed aggressive squamous cell carcinoma of head and neck cases in combination with platinum in 2011 [29-31]. During this time, John Mendelsohn also continued his interest in various mechanistic aspects of EGFR's inner working in cancer cells, largely through collaborative studies with his colleagues (Figure 2C).

Dr. Mendelsohn's presidency will be credited for an enormous broadening of MD Anderson's footprint in basic and translational research programs, initiating multidisciplinary research programs, uplifting the value of team science, and creating theme-centered new research institutions (including the Red and Charline McCombs Institute for the Early Detection and Treatment of Cancer, the Duncan Family Institute for Cancer Prevention and Risk Assessment, the Sheikh Khalifa Bin Zayed Al Nahyan Institute for Personalized Cancer Therapy and advance personalized medicine, where he was the co-director from 2012 to 2018). Similarly, Dr. Mendelsohn's also provided his visionary leadership to the educational mission of MD Anderson and its effective integration into Texas Medical Center, and thereby made MD Anderson a degree-granting institution in several disciplines. MD Anderson's history books will remember John Mendelsohn as someone who created an unprecedented fabric of administrative, scientific and fiscal resources. In brief, John Mendelsohn leadership has changed for the better the bench marks of excellence for betterment in core values of MD Anderson and other institutions he served.

## REFLECTIONS

To spotlight John Mendelsohn's influence as a fine human being, as an administrator and as a scientist, here we provide personal reflections of John from us and several colleagues. It is our hope that these reflections, some dating back to 1971, will shed light on John Mendelsohn's caring personality and visionary outlook. Another opportunity to learn more about Dr. Mendelsohn's career and achievements from his point of view is provided by the Oral History Project at MD Anderson's Research Medical Library, which has approximately 6 hours of recorded interviews with him, organized and indexed for easy access [32].

### Rakesh Kumar

I was one of those who were fortunate to be John Mendelsohn's mentee, collaborator, colleague and life-long friend. During these years, John Mendelsohn's persistence in thinking about how science can improve the lives of cancer patients was unwavering. He was compassionate, caring, humble and a remarkable professional and person. He will be remembered equally for his generosity to his patients, colleagues and friends as he will be for his impactful contributions to numerous cancer centers. John Mendelsohn was a brilliant cell, tumor and molecular biologist equipped with hard core biochemistry.

Since the time I first met John Mendelsohn in June 1988 and last visited him in late June 2018, I have always found him full of scientific curiosity and always driving our scientific discussions towards the next big conceptual questions. A scientific discussion with Dr. Mendelsohn was always an intellectual feast. In addition to sharing a very exciting academic journey with Dr. Mendelsohn, I also had the privilege to be part of the President's Research Strategy and Advisory Committee (RSAC) at MD Anderson for several years. This was a wonderful learning experience for me to witness first-hand Dr. Mendelsohn's leadership in all matters of importance. It is without a doubt that Dr. Mendelsohn was the best professional mentor I could have asked for, and I will sorely miss him and our conversations together.

### Ferid Murad

John Mendelsohn was a very effective and respected administrator and an outstanding scientist who was on the forefront of targeted cancer therapy with his antibody to epidermal growth factor kinase. He also led the MD Anderson to become the world's leading cancer hospital. More importantly he was a very good friend who helped many of our family members and friends with their cancer treatment at MD Anderson by facilitating theirs visits and treatment with appropriate oncologists. We shared faculty for some of our joint committees between the MD Anderson and the University of Texas-Houston. John was on the committee that appointed me as the Director of the Institute of Molecular Medicine. We joined Houston within months of each other and rapidly became good friends. The Oncology field will suffer a very big loss with his passing away.

### Oliver Bogler

John Mendelsohn's vision of MD Anderson's mission went beyond Texas and the USA, to encompass the world. He created the Center for Global Oncology which provided different ways that cancer-fighting institutions across the world could engage with MD Anderson. I was fortunate to be a part of the academic component of the Center, Global Academic Programs, which focused on building a network of Sister Institutions, which were like-minded academic centers with significant cancer programs [28]. It was John Mendelsohn's vision that MD Anderson should not only be advancing research and care but should, as part of its education mission, work to share its expertise with global partners that drove our work. During John Mendelsohn Presidency, MDACC created a network of Sister Institutions grown to 20 member institutions with significant cancer programs and like-minded academic approaches [25]. The network had, at its peak, 35 members from 25 countries, who came together for collaborative research projects, published hundreds of papers together, exchanged training scientists and physicians, and met annually for an educational conference either in Houston or hosted by a Sister with around 800 attendees. I was lucky to be able to accompany John on visits to some of our Sisters, and was inspired by him every time: he brought his spirit of inquiry, his leadership talents and his generosity to this work and it was this that energized the work.

### Bert W. O'Malley

John Mendelsohn was the archetypical ‘man for all seasons’. As a friend, I saw him as a gregarious and compassionate gentleman who handled himself always with intellect and class. Few scientists or administrators can claim the variety of key jobs and discoveries that are part of John's sterling record. First and foremost, he was a medical doctor who cared about his patients and people in general, and who valued and promoted the successes of his trainees. This was evident throughout his career. His role in developing an early and successful targeted therapy via EGFR antibodies that stimulated further innovative and similar approaches in the field of oncologic therapy was perhaps his singular greatest scientific achievement; it also was a treatment from which I personally benefitted during my recovery from throat cancer some years ago. Perhaps this remains as his greatest long-lasting gift to mankind.

John also excelled in another sub-vocation as an outstanding academic and hospital administrator. He contributed his vision and judgement to development at the University of California San Diego, Memorial Sloan-Kettering, and the jewel of his crown, the University of Texas M.D. Anderson Center in Houston. It was there that I observed first-hand his ability to build, recruit, and develop what many think is the greatest oncology center in the world. His scientific focus was on translational research, and the movement of new laboratory discoveries into clinical trials. This was again, consistent with his final medical goal as a physician - the delivery of first class and innovative care to the oncology patient. Although he was awarded numerous honors and scientific distinctions, I know that in addition to his family and many close friends, he valued most his scientific contributions to oncologic pharmacotherapy and his leadership in the spectacular growth and success of the M.D. Anderson Hospital and Cancer Center.

### Gabriel N. Hortobagyi

Reflexions on John Mendelsohn - An outstanding scientist and academic leader, John succeeded for another reason: his humanity. John had a remarkable ability to connect on a very personal level with people: his colleagues, his friends and family, employees at the institutions he led and community members with whom he interacted during fundraising, enjoyment and support of the arts and other endeavors. When you spoke with John Mendelsohn, you had his undivided attention. He was genuinely interested in your opinions, your occupations and your interests. Whether you were a fellow scientist or a total stranger, John had the innate ability to connect. His open smile, his firm handshake, his self-effacing modesty, all contributed to this connection. Whether you met John in his presidential office, at a meeting of the Board of Visitors, at the Opera, at a restaurant or during travel overseas, John was always the same: warm, friendly, open, positive, optimistic and caring. I had many discussions with John about our institution, our shared passion for research and progress, our focus on the welfare of our patients and the commitment to problem solving. John was an outstanding servant leader. As a department chair, I could get a hold of John by calling his office at any time. Whether he was in town or traveling, he would get back to me within an hour. He had many important issues on his mind, but when you needed him; your problem would become number one at that moment.

We also shared many moments of joy at the Houston Grand Opera, the Paris Opera, and the Metropolitan Opera in New York or at a number of opera houses around the world. His passion for music was deep and his contributions to HGO during his tenure as Chairman of the Board were many. We also shared our love for visual arts. We both collected paintings and were excited about discovering new artists and new works. John was proud of Anne Mendelsohn's great work as member of the Board of Directors of the Houston Museum of Natural Sciences and we often spoke about the amazing transformation of that museum thanks to her involvement and leadership. We enjoyed fine dining and exploring fine wines together, whether in Houston or during our travels through France or Italy. We lost a great leader in science with his death, but even more importantly, we lost a great human being and a friend we will forever miss.

### Tony Hunter, Salk Institute

I first met John shortly after I arrived at the Salk Institute to do postdoctoral studies with Walter Eckhart in 1971. Our paths crossed early on, because at the time John was working on DNA replication in lymphocytes with Mehran (Mickey) Goulian at UCSD, while we were using polyomavirus as a model system to define the molecular mechanisms of DNA replication. Indeed, John had a paper in Nature New Biology in late 1973 describing the presence of RNA in nascent DNA from lymphocytes, and a few months later we reported that short chain (Okazaki) nascent DNA fragments derived from replicating polyomavirus DNA also had RNA at the 5’ end, which turned out to be the primer for discontinuous lagging strand synthesis. After I returned from the UK in 1975 as an Assistant Professor at the Salk, our paths continued to cross although not so frequently, partly because the UCSD Cancer Center, where John was the founding director, was located downtown. However, our scientific paths intersected again in 1980, after we discovered tyrosine phosphorylation as a new protein modification, and Stanley Cohen followed our finding to show that the EGF-stimulated EGF receptor kinase activity is tyrosine specific. At the time, Gordon Gill, a kinase maven who had been at UCSD since 1969, had also begun working on the EGF receptor kinase, and at the same time as us in 1981 reported that EGF stimulation of A431 cells caused a rapid increase in tyrosine phosphorylation of cellular proteins. John and Gordon Sato knew Gordon Gill and his work on the EGF receptor, and this sparked their interest in generating monoclonal antibodies that recognized the EGF receptor, which they reported in their 1983 PNAS and Mol Biol Med papers. Amazingly, one of that first set of mAbs, clone 225, ultimately became the cetuximab drug after it was humanized.

Our paths crossed many times after John left UCSD, often at scientific meetings, or when I visited MSKCC or MDACC to give a talk. John and I were also co-recipients of the Bristol-Myers Squibb Cancer Research Grant Award in 1997, and we served together on the BMS Cancer Award committee for the next three years, with the task of selecting the winners of the annual BMS Award for Distinguished Achievement in Cancer Research, which I am pleased to say John himself won in 2004. I was delighted to learn in May of 2018 that I would share the 2018 Tang Prize in Pharmaceutical Science with John and Brian Druker for our work on targeting tyrosine phosphorylation for cancer therapy. It was very sad that John was too ill to attend the ceremony, but he lived long enough to know how much his seminal work on developing an EGFR antagonist monoclonal antibody was appreciated by the scientific community, and above all the grateful patients who have benefited from cetuximab.

### Robert A. Weinberg, Massachusetts Institute of Technology

Others have described at length John's contributions to clinical therapy, but I would like to cite another side of him. He was an extraordinarily fine human being. Gracious, unfailingly generous and invariably self-effacing. We might imagine that these traits of personality were peripheral to the main trajectory of his multi-faceted career, but to my mind they were central. They allowed him to attract colleagues and build institutional cultures that succeeded in generating the extraordinary benefits for cancer patients that flowed from the institutions that he led during his long career.

### Robert C. Bast, MD Anderson

John Mendelsohn was not only a great physician, scientist and internationally-respected leader, but an exceptional human being who loved and enjoyed his family, friends, tennis, history and opera. His enthusiasm, energy and optimism were infectious.

John's achievements have been an inspiration. Discovery and development of cetuximab has become one of the classic examples of translational oncology and precision therapy. Perhaps his greatest achievements have been in establishing and strengthening centers of excellence in cancer care and research, initially at University of California at San Diego as its founding director, then at Memorial Sloan-Kettering as head of medicine and most notably at MD Anderson. During his 15 years as president of MD Anderson, John took a great institution and grew it into arguably the best cancer center worldwide, caring for more than a hundred thousand patients, evaluating the majority of FDA-approved oncologic drugs and making significant contributions to multiple areas of cancer research. While MD Anderson had long been known for its patient care and clinical trials, John strengthened translational research and created an optimal environment to develop the careers of both physician-scientists and clinician-investigators. During his tenure, the facilities for patient care and research were expanded dramatically, spreading to a second campus south of the Texas Medical Center. MD Anderson began to partner with hospitals throughout the country and established a research network with 35 institutions in 25 countries world-wide. Growth of MD Anderson was facilitated both by increased clinical activity and by an expansion of philanthropy. John and Ann Mendelson were the perfect team to engage supporters in the Houston community and throughout the nation.

Having served as cancer center director at UCSD, John was attuned to the priorities of MD Anderson's CCSG “Core Grant” that is required for our recognition as an National Cancer Institute-designated cancer center. The grant is renewed every five years and generally involves several thousand pages describing all of MD Anderson's research and the shared facilities that support that research. This is one of the few NCI grants that require a site visit by a team of more than 20 physicians and scientists from other US centers to provide a critical evaluation of how well the center is functioning and whether it is worthy of NCI designation. At the conclusion of a day of presentations, there is an executive session with the site visitors and only the cancer center director and his deputy. At the conclusion of the Executive Session for John's last site visit as cancer center director and president, after John's last comments the presumably critical site visitors broke into applause, a unique tribute to a great leader. All of us who knew John Mendelsohn miss him deeply, but we can only applaud his leadership and his example.

### Raymond Sawaya, MD Anderson

When John Mendelsohn was announced as the selected candidate to be our Third President in 1996, the news was received by many at MD Anderson with skepticism. This obviously did not deter John from moving forward with his Vision and style, and maybe it even emboldened him in doing so. In the end, his 15 year tenure as President, resulted in an explosive growth in our clinical and research programs, in our facilities and operating budget, and these were matched with our rise in National rankings to the number one spot in the country.

I was fortunate to be asked to serve on John's Management Committee for two years, and that gave me a closer inside look into his leadership and managerial styles. From these observations, it became obvious that John was a great listener and a consensus builder, and that he would not shy away from implementing bold ideas. In fact, I was a direct beneficiary of such an endeavor when he agreed to let me serve simultaneously as Chair of the Department of Neurosurgery at Baylor College of Medicine, an Institution in the Texas Medical Center that many here viewed as a direct competitor to our own Institution. For John, it was the desire to lend a helping hand to a distinguished Institution during a period of hardship in their history, and he viewed my role as that of an Ambassador helping bridge Institutional divides, and strengthening collaborations. With the perspective that we have gained over these years, MD Anderson, and all of us affiliated with this great Institution, were most fortunate to have had John Mendelsohn as our Leader.

### David M. Gershenson, MD Anderson

During most of John Mendelsohn's tenure as President of MD Anderson Cancer Center, I had the privilege of serving as Chair of the Department of Gynecologic Oncology. John had a major influence on two aspects of my professional life. Under his leadership, the department experienced unprecedented growth from 10 to 31 faculty members; we significantly enhanced our translational research activities; and our faculty played an integral role in garnering two SPORE grants (Ovarian Cancer and Uterine Cancer), a T32 Training grant, and a Nanomedicine grant. On a more personal note, John served as a role model in my research career as I led the NRG Oncology Rare Tumor Committee in the development of targeted therapy trials for chemoresistant rare ovarian cancers.

### Christopher J. Logothetis, MD Anderson

I met John Mendelsohn when I was a midcareer oncologist, certain I had the answers and more than willing to voice them. John, among many, taught me otherwise! In his way, he listened, showed alternatives, and often firmly redirected. Over the years we learned to understand our opposing perspectives, confident that we shared a common goal. The path from mentee to colleague and friend was rich with lively debate, eased by a shared passion for literature and history. John will be remembered as a member of the fraternity whose presence left a positive mark on the world. In this tumultuous time in academic medicine, we would be wise to take a page from his life's playbook.

### Stanley R. Hamilton, MD Anderson

We are fortunate in academic medicine to have the occasional opportunity to work with a “rainmaker” who innovates a field that translates into long-term and broad positive impact for patients. John fulfilled that role in targeted therapy for cancer patients and was also an insightful and skilled executive administrator as well as a true gentleman.

### Mien-Chie Hung, China Medical University, Taiwan

Dr. Mendelsohn was a visionary leader! Under his leadership, MD Anderson Cancer Center was constantly ranked as number one cancer center in the US, and in my personal opinion, it is a number one cancer center in the world. He is my role model. While he took the president position at MD Anderson, he told us that his project is “MD Anderson”! I served for more than three decades in MD Anderson, and was fortunately to be in a leadership role under Dr. Mendelsohn for more than 15 years. After I became President at China Medical University in Taiwan in February 2019, I also told myself my project is “China Medical University”!!! I missed Dr. Mendelsohn's friendship, mentorship, and leadership enormously. We certainly lost a giant in our cancer community.

### George M. Stancel, University of Texas Health Science Center Houston

Most people know about Dr. Mendelsohn's research and leadership activities, but may not know his commitment to education that I saw when I became the dean of the University of Texas Graduate School of Biomedical Sciences (GSBS) at Houston in 1999. M.D. Anderson faculty were major contributors to MS and PhD training, but degrees were awarded solely by the University of Texas Health Science Center since Anderson did not have degree granting authority at that time. Dr. Mendelsohn, with the concurrence of Dr. Willerson, the president of the Health Science Center, asked me to arrange for the degrees to be awarded jointly by Anderson and the Health Science Center. This a huge task that would require the Texas Legislature to change the state education code, so I asked him why. His answer both surprised and impressed me – “If M.D. Anderson's name is on the diploma, graduate education becomes a formal institutional mission and I can demand and reward excellence from our faculty who participate.” GSBS received the required authority in 2001 but that wasn't enough for Dr. Mendelsohn – he wanted Anderson to be accredited as an independent degree granting academic institution by the Southern Association of Colleges and Schools (SACS) – another huge undertaking! With his support and that of the Health Science Center this was accomplished in 2005, and in 2010 the name of the graduate school was changed to The University of Texas MD Anderson Cancer Center UTHealth Graduate School of Biomedical Sciences. The school is now a full partnership between the two institutions that I believe is a model for institutional cooperation to provide outstanding, and cost effective, graduate training. This brief description illustrates that John Mendelsohn deserves credit and recognition for his vision and leadership in biomedical education as well as his outstanding contributions in research, mentoring, and institutional and professional leadership.

## CONCLUDING REMARKS

John Mendelsohn's vision for helping cancer patients was far ahead of his time and driven by his core leadership principles. He believed in finding the most impactful means to translate scientific advances from the bench to the bedside in order to deliver the best possible cancer care. Dr. Mendelsohn unique leadership skills allowed him to be an effective administrator in this regard and were aided by his natural instinct to foresee emerging science-driven trends. These instincts allowed John Mendelsohn to grow numerous cancer centers to serve cancer patients.

John Mendelsohn pioneering work on interrupting the growth factor receptor signalling will continue to serve as a guidepost in Cancer Medicine, and will inspire the development of the next generation of RTK-directed targeted therapeutics. During his illustrious career over a span of nearly half a century, John Mendelsohn touched a large number of colleagues, oncologists, cancer biologists and students, provided care to a large number of cancer patients, and contributed to the growth and upward trajectories of the institutions in which he worked. It is certain that John Mendelsohn's contributions on scholarship, patient care, and institutional growth will be felt for a long time to come. Dr. Mendelsohn summarized his professional journey: “Long term, I will work hard to complete my life long story of my 81 years of learning and dealing with the exciting experiences, watching chemo therapy develop from non-existing treatment to a very successful current approach to cancer cure or prolongation of life.” – John Mendelsohn, M.D., January 16, 2018 (personal communication).
